# FoodChain-Lab: A Trace-Back and Trace-Forward Tool Developed and Applied during Food-Borne Disease Outbreak Investigations in Germany and Europe

**DOI:** 10.1371/journal.pone.0151977

**Published:** 2016-03-17

**Authors:** Armin A. Weiser, Christian Thöns, Matthias Filter, Alexander Falenski, Bernd Appel, Annemarie Käsbohrer

**Affiliations:** Department Biological Safety, Federal Institute for Risk Assessment (BfR), Berlin, Germany; Health Protection Agency, UNITED KINGDOM

## Abstract

FoodChain-Lab is modular open-source software for trace-back and trace-forward analysis in food-borne disease outbreak investigations. Development of FoodChain-Lab has been driven by a need for appropriate software in several food-related outbreaks in Germany since 2011. The software allows integrated data management, data linkage, enrichment and visualization as well as interactive supply chain analyses. Identification of possible outbreak sources or vehicles is facilitated by calculation of tracing scores for food-handling stations (companies or persons) and food products under investigation. The software also supports consideration of station-specific cross-contamination, analysis of geographical relationships, and topological clustering of the tracing network structure. FoodChain-Lab has been applied successfully in previous outbreak investigations, for example during the 2011 EHEC outbreak and the 2013/14 European hepatitis A outbreak. The software is most useful in complex, multi-area outbreak investigations where epidemiological evidence may be insufficient to discriminate between multiple implicated food products. The automated analysis and visualization components would be of greater value if trading information on food ingredients and compound products was more easily available.

## Introduction

Worldwide, foodborne illness causes billions of dollars in healthcare related costs each year [1], and more in economic losses to farmers, distributors and food retailers [2,3]. In the case of foodborne disease outbreaks, rapid identification of the causative food product is essential, as the medical and economic damages incurred increase with the duration of the outbreak. Outbreak investigations often begin with interviews of cases about consumption of food products and may proceed to an analytical study such as a case-control study [4]. These epidemiological analyses are paralleled by microbiological investigations of implicated food products. Authorities may need to reconstruct relevant food distribution networks to identify a causative food product. Distribution network reconstruction may be time-consuming and labor-intensive as information has to be collected from each company involved in the affected product chain. IT-based traceability solutions, which support the tracking of food items in food business operations and which have existed for several years [5], are not currently mandatory. Companies may store data in arbitrary formats, including non-digital formats, making compiling and analyzing data difficult. Information on food deliveries may be collated by different public authorities, leading to data errors; for example, different or misspelled names may be assigned to the same company or product. Global trade increases the complexity of many food product networks. Simple food chain structures may be delineated manually, but in outbreaks with complex food chain network structures, a manual network reconstruction approach relying on the “one step back, one step forward” principle specified in Regulation (EC) No 178/2002 [6] may not be applicable. Companies under investigation may not have retained all necessary information. Delineating separate network structures for each supply chain pathway in a complex distribution network may not be feasible and the technical infrastructure to handle large volumes of data may not be available.

During an outbreak of Shiga toxin-producing *Escherichia coli* O104:H4 in Germany in 2011, the Federal Institute for Risk Assessment (BfR) initiated development of the open-source software tool “FoodChain-Lab” to support trace-back and trace-forward analysis of implicated feed or food items along supply chains. Sprouts produced by a horticultural farm in Lower Saxony were identified as the vehicle for the pathogen; a specific lot of fenugreek seeds imported from Egypt was the most likely source of contamination [7].

FoodChain-Lab has subsequently been applied in other outbreak investigations, including a 2012 outbreak of norovirus gastroenteritis in Germany, in which a total of 390 facilities, mostly schools, were affected with 10,950 registered cases of gastroenteritis, including 38 hospitalizations. Epidemiological and trace-back investigations showed that a consignment of frozen strawberries from China was the source of the outbreak [8]. An increase in hepatitis A cases was observed in northern Italy in 2013 resulting in another investigation managed by the European Food Safety Authority (EFSA), which invited BfR to scientifically support it by making use of FoodChain-Lab [9,10].

The objective of this research was therefore to create a free and open-source software resource for public health experts, capable of supporting investigations of supply chains as well as exposure and risk assessments in outbreaks. Specifically, the software developed *ad hoc* during the German EHEC outbreak in 2011 [7] has been redesigned with new features facilitating efficient handling and analysis of food trade data, as illustrated here with examples.

## Methods

### Definitions

Traceback analysis aims to identify the production and distribution chain of a product suspected on epidemiological or microbiological grounds to be the vehicle of a food-borne outbreak. Within this article we make use of the following definitions:

- A **station** represents a point in the supply chain that is sending or receiving a food product; for example, a company producing or distributing a food product or a person consuming it
- A **trace** is a path along the supply chain a contaminated food product may have taken; it may include mixing and splitting events
- A **case** is a station or a lot where the pathogen causing the outbreak arises. The definition of a case is outbreak specific. It may be defined as a minimal number of persons affected at a certain station by having specific disease symptoms, a confirmed pathogen and/or consumed a food product during a defined period. A lot with a confirmed pathogen can also be defined as a case. For example, a case can be a school within a certain region having at least 10 sick pupils showing identical symptoms in a certain time frame.

### Data Management

#### Data Integration

FoodChain-Lab offers two options to collect and import data into an internal database:

1. Automatically generated semi-filled Excel templates that can be sent to local authorities for data completion and afterwards be imported into the database
2. A direct interface to the database for manual data input

Once the data has been successfully transferred into the database it can be validated, corrected and updated. A logger function ensures that all modifications applied to the data are tracked automatically.

**Fig 1:**
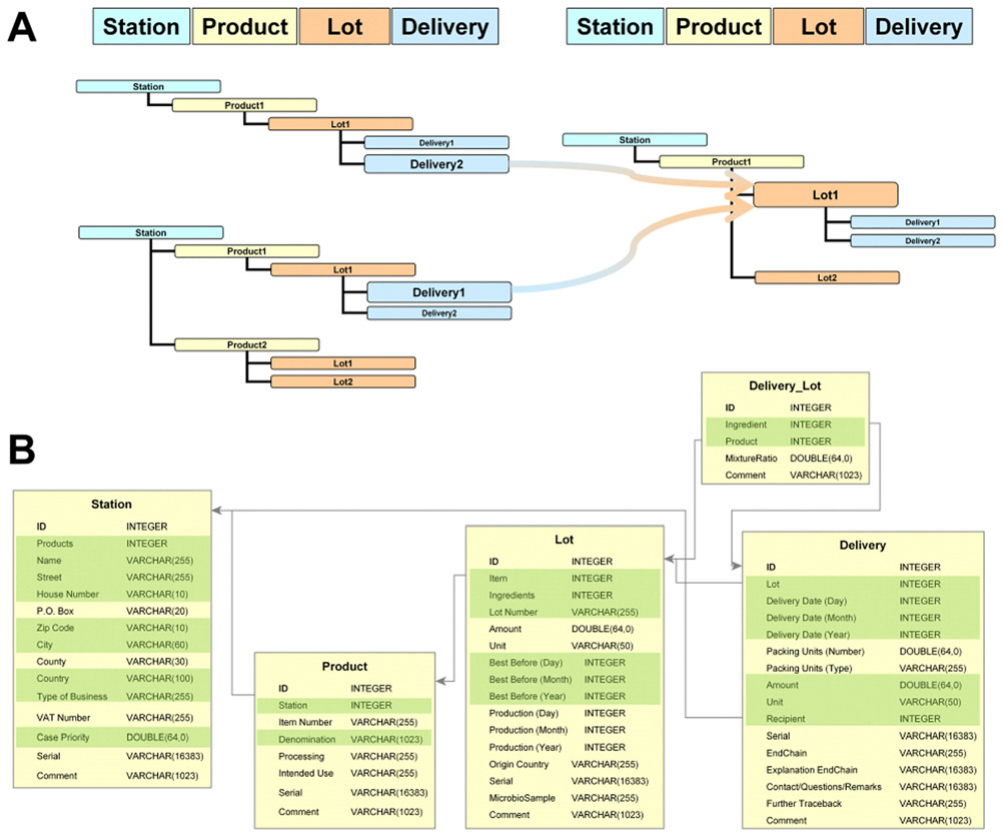
Schematic description of the data structure in FoodChain-Lab. A) General principle of food supply chain reconstruction as performed by FoodChain-Lab. The connection between a delivery to one station to the lot of the following station is of major importance for tracing analysis. B) Detailed data structure used by FoodChain-Lab for storing food supply chain information. The most important attributes for detailed data analysis are highlighted in green.

The integrated database has been specifically designed to meet general requirements of food and feed chain networks enabling the user to store all relevant information in a structured way on the basis of four main entities: STATION, PRODUCT, LOT and DELIVERY. Fig 1A provides the principal schematic description of the data structure as it has been implemented. The predefined data structure of the database allows detailed information to be attached to each of the entities (Fig 1B).

An important feature of the database structure is that it is capable of storing supply chains of indefinite length and arbitrary complexity. Moreover products can be “produced” in varying numbers of intermediate steps and be derived from one to any number of ingredients. This allows for food chains where parts of a batch are handled by several intermediate distributors.

#### Safeguarding Data Quality

In order to perform meaningful analyses of food or feed tracing network data it is crucial that the highest possible data quality is ensured, which is a major challenge in most outbreak situations. FoodChain-Lab supports this task by performing plausibility checks on any collected data, ensuring that information is consistently integrated into the database. It further provides functionalities for similarity searches on the levels of all entities applying the Levenshtein distance measure [11] with user-defined thresholds. This function is especially important for identifying stations that have been falsely registered as independent entities, since these types of errors significantly hamper the success rate of any tracing analysis. In addition, to ensure data consistency, the system verifies that subsequent deliveries follow each other chronologically and that in- and out-amounts of a lot at a station are plausible.

The system further provides information on whether all products have been completely traced back, e.g. to a specific business type such as primary producers, and for which stations delivery information is still missing.

### Visualization

A major application of FoodChain-Lab is the analysis and visualization of food tracing information. This is accomplished by the construction and visualization of interactive food chain network graphs. A network graph in general consists of nodes and edges, where edges always connect two nodes. When applied in the field of food tracing, a network graph comprises of edges representing food product transportation events and nodes representing stations. This feature is realized in a KNIME node called “Tracing View”.

To visualize the network graph in an aesthetically pleasing way and allow the user to immediately identify supply chain contexts, the Java Universal Network/Graph Framework (JUNG) [12] with its force-directed graph drawing algorithms was integrated. The Fruchterman-Reingold algorithm [13] has proved to be useful in most outbreak scenarios.

Stations and deliveries can be assigned individual colors and shapes based on their properties, as detailed in the documentation.

**Fig 2:**
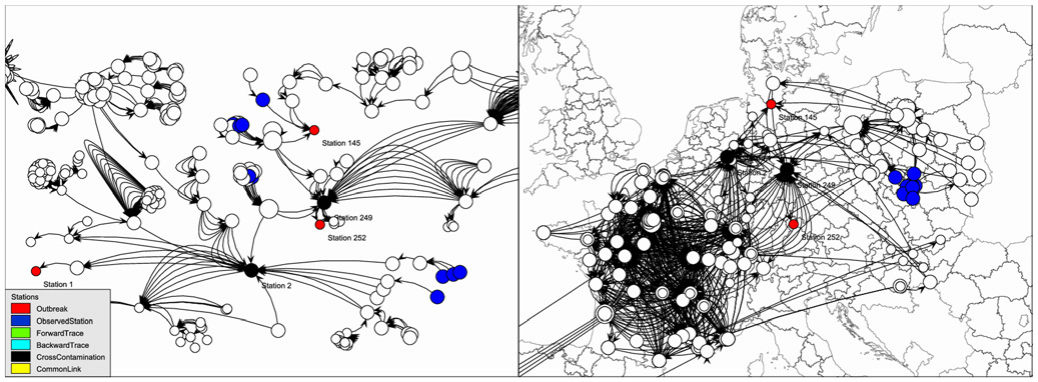
Simple basic visualization combining network and geographical view. Interactive trade network visualization: network graph (left) and GIS map view based on shapefiles (right). Stations on the left and on the right are identical and always synchronized, i.e. the blue stations are identical. In contrast to the GIS view, the graph view automatically groups stations that are connected via deliveries to demonstrate relationships between stations. This figure can be reproduced by using the available sample data.

In addition to network graphs a geographical map view of the data is provided (Fig 2). The geographical view is based on shapefiles [14] or on maps from OpenStreetMap [15]. If geographical information, i.e. latitude and longitude data, is not present the system is able to generate it from address information associated with stations. This is done by using a geocoding service, e.g. MapQuest [16] or the open-source project Gisgraphy which allows in-house geocoding of confidential data [17].

All visualizations are interactive, adapting immediately to any change in configuration or simulation results. All data, calculation results and visualizations can be exported as data tables or images for use in other KNIME nodes (e.g. data views, data manipulation, statistics and reporting), other tools or in reports. All data analysis steps and workflows can be saved including all intermediate calculation results and shared with other investigators.

### Analysis features

#### Observing traces

**Fig 3:**
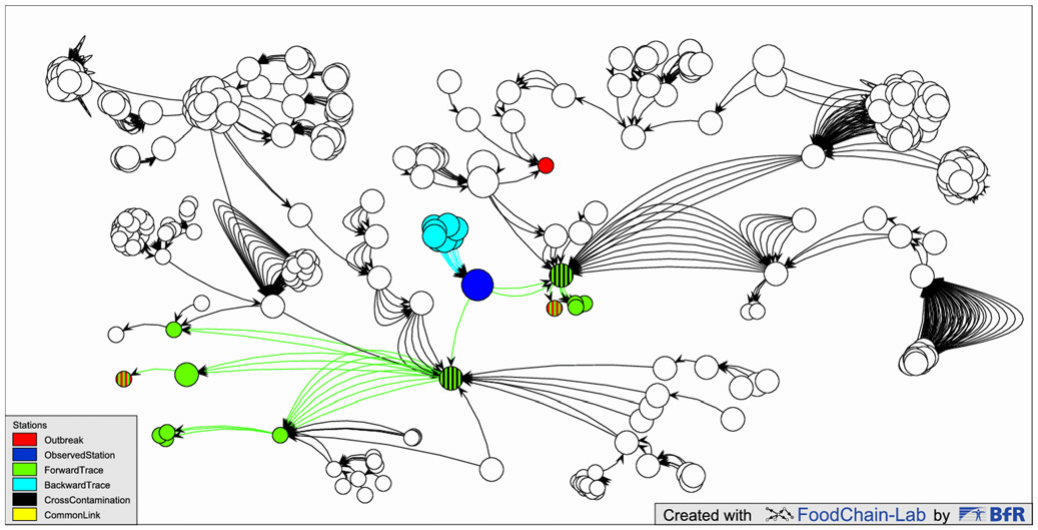
Interactive visualization of the complete trace of an observed station. Outbreak stations are marked in (striped) red. After selecting a station to be “observed” (blue) the view immediately shows the whole trace of that station: the green/green striped stations and deliveries indicate the forward trace and turquoise denotes the backward trace. In the two stations receiving deliveries from the blue station, cross-contamination is also assumed (black stripes). Deliveries leaving the observed station arrive at two outbreak stations (red/green), but not at the third one (red). This figure can be reproduced by using the available sample data.

This feature allows for analysis of the whole forward and backward trace of a user-defined set of stations/deliveries. For example, the user can interactively select an implicated station to show all stations/deliveries on its forward and backward trace (Fig 3).

#### Cross-contamination

Cross-contamination, which may happen at a station between different lots, can be simulated at station level and fine-tuned at lot level. This feature directly changes the traces of the products and the paths calculated for a contamination.

#### Stop-contamination

This feature allows simulation of inactivation of agents during processing steps, e.g. norovirus would probably not survive an ultra-heat treatment. This feature alters the traces of all associated food items as well.

#### Tracing Score

A key request on the software is the search for a common station or delivery that connects all defined cases. FoodChain-Lab automatically computes a “tracing score” for all stations and deliveries. The higher the score of a station or delivery, the more likely a contamination of a commodity at a specific station or delivery can explain the outbreak. The score cannot be interpreted as a probability that contamination has taken place at a specific station. But it is correlated to the probability that a contaminated product has passed through the station and may help to identify a common contamination source.

The tracing score is calculated using the following formula:

$$\text{Score}\left(s_{i}\right)=\frac{\sum_{j=1}^{n}w_{j}t_{ij}}{\sum_{j=1}^{n}w_{j}}$$

Where

- s_i_ is the i-th station or delivery
- w_j_ is the weight of the j-th station or delivery as defined by the investigators. Usually, it has a value of 1 for confirmed cases, a value of 0 for no evidence on contamination and a value in between for suspicious stations or lots
- t_ij_ has a value of 1, if there is a trace from s_i_ to s_j_ and a value of 0 otherwise
- n is the total number of stations and deliveries

The value of the tracing score Score(s_i_) is between zero and one.

The visual size of a station in the TracingView is linked to this score resulting in bigger nodes for those stations more likely to be relevant in the outbreak investigation compared to smaller ones.

#### Regional Analysis

There can be regional relationships between stations, e.g. because of staff working at more than one company within a region, agricultural products grown in the same area or environmental influences such as the use of irrigation water from a common source.

**Fig 4:**
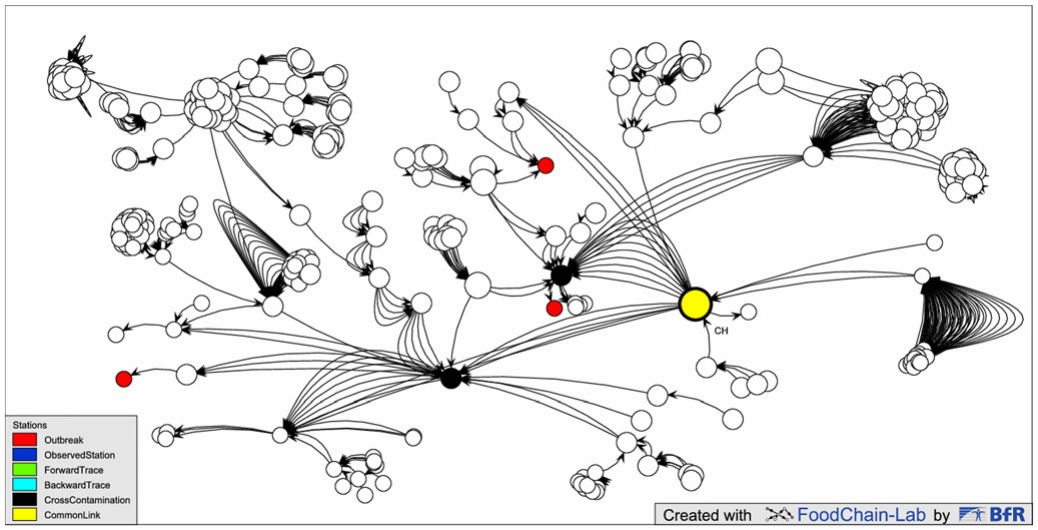
Collapsing many stations into one meta-station. The meta-station is a collapsed version of all stations from a certain country (collapse based on attribute “Country”) resulting in a common link (yellow), i.e. this collapsed station (the country) has traces to all outbreak stations (red) and therefore carries the maximum score of 1. This figure can be reproduced by using the available sample data.

FoodChain-Lab supports this type of analysis by enabling the merging of selected stations into a meta-station that is treated as a single station for the analysis (Fig 4). Merging can be done using

- manual selection in the geographic view
- common attributes of a station, e.g. zip code
- clustering algorithms for geographical coordinates: k-means clustering [18] or DBSCAN [19].

This feature proved to be very useful, especially when the data are complex or when data gaps may exist at e.g. the primary producer level.

### Software Architecture

FoodChain-Lab has been implemented as a modular extension to the open source data analytics platform Konstanz Information Miner (KNIME) [20]. KNIME enables visual assembly of data analysis workflows. These workflows consist of so-called nodes and edges. Each node is able to perform a specific data processing task while edges define how information flow is directed between nodes. Each node contains an extensive node description, explaining node functionality and the node-specific user interface in detail.

All data analysis functionalities of FoodChain-Lab have been implemented as KNIME nodes written in the Java programming language.

**Fig 5:**
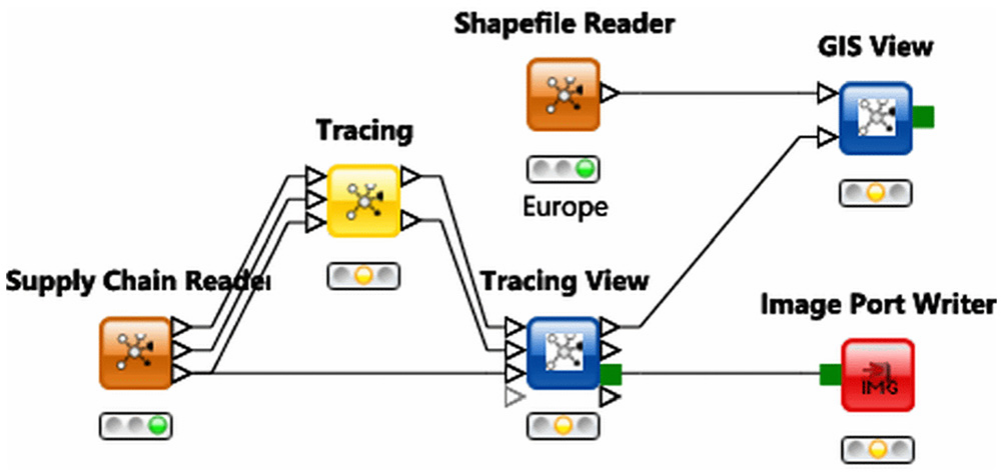
KNIME workflow “Tracing and Visualization”. The “Supply Chain Reader” node is used to read tracing information from the integrated FoodChain-Lab database. Data processing results are then provided to the “Tracing” node, which is able to perform specific tracing calculations. The “Tracing View” node is the main node for interactive data and tracing analysis. Finally the “GIS View” node can be used to create a geographical visualization of the food chain network using additional GIS information fed in via the “Shapefile Reader” node.

Fig 5 provides an illustrative example of a FoodChain-Lab data analysis workflow using nodes from the FoodChain-Lab node repository as well as regular KNIME nodes.

A major advantage of using the KNIME platform is the multitude of existing data analysis features. This allows for performing tasks in many related areas such as microbial risk assessment and also for integration of results into workflows using other community node libraries.

FoodChain-Lab is free and licensed under the GNU General Public License. The installation guide, example workflows, video, tutorials, sample data, example scenarios and detailed descriptions on all features are available at https://foodrisklabs.bfr.bund.de while source code and a ticket system are hosted at GitHub.

FoodChain-Lab runs on all common operating systems: Windows, Linux and Mac OS X.

## Results of Evaluation

During the EHEC outbreak in Germany in 2011 [7] it became evident that no suitable analytical tool was available to public authorities for handling tracing data. The state of the art was to carry out tracing analysis manually with existing tools and skills. Other specialized tools and databases [21–24] are often not accessible to outbreak investigators and are designed for purposes relating to specific data structures, attributes or supply chains. Furthermore, the challenge of integrating information from small companies, which may not have an computerized inventory management system to comply with directives on tracing, was not addressed [25].

We here summarize the key findings of applying the software FoodChain-Lab during two real world outbreak investigations.

In **2011** the Task Force **EHEC** considered two different analytical strategies aiming to identify the source of contamination: “manually”, by checking if an implicated sprout producer had supply chains to all cases, and “IT-based”, a lot-based backward tracing from the cases resulting in a reconstruction of the real flow of all food items consumed in common dishes within outbreak clusters. The first approach was quickly successful as it became clear that a specific sprout producer had connections to nearly all outbreak stations. The backward tracing approach was successful as well, but leading to another result—fenugreek seeds from Egypt were identified as the contaminated food commodity causing the outbreak.

In general, the advantage of the first approach is that it leads to results quickly but has some disadvantages: firstly, there is a risk of expectancy bias; secondly, it does not reconstruct supply chains in terms of individual lots, providing less detailed evidence; and thirdly, it is most applicable when a simple supply chain is of particular interest.

**Fig 6:**
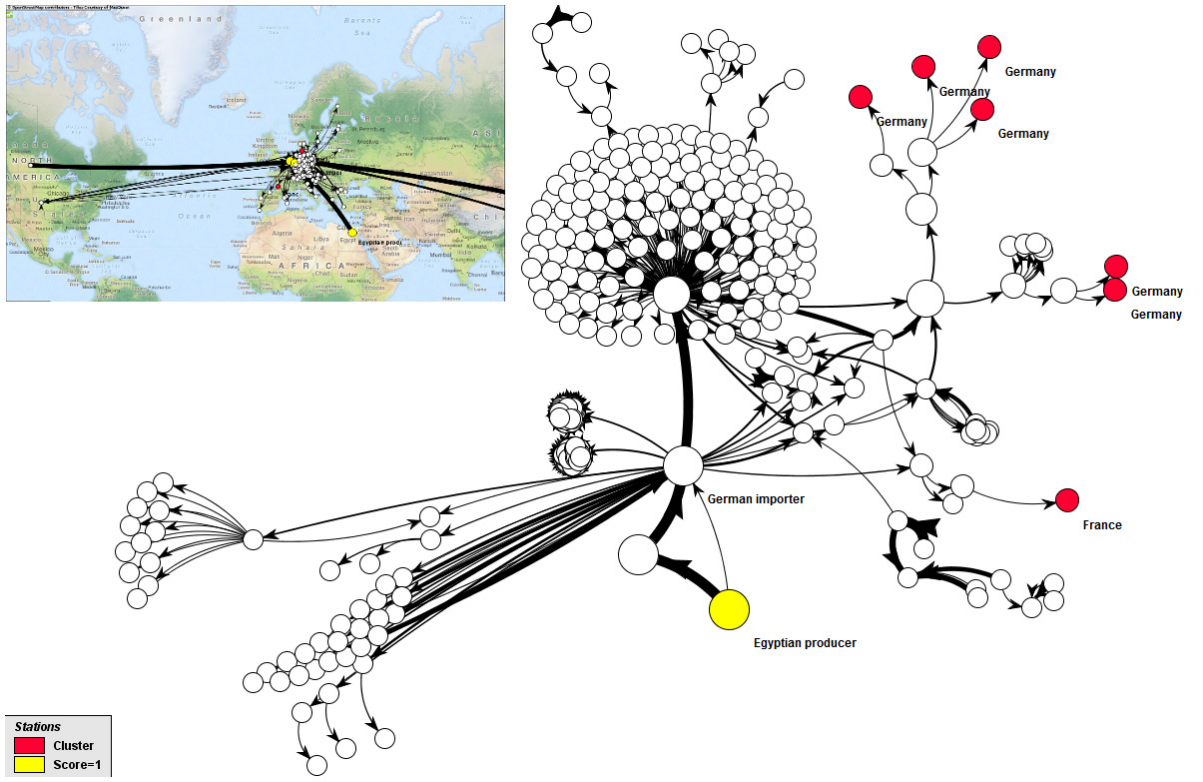
EHEC outbreak 2011. All seven outbreak clusters in Germany and France (red) investigated in detail can be traced back to the source of the outbreak, which is the producer of fenugreek seeds in Egypt (yellow, the backtrace score calculated is 1). Numerous other stations without cases (no color) also received seeds from this producer. The automatically generated network view is synchronized with a geographical view based on maps from OpenStreetMap.

In the case of the EHEC outbreak, supply chains were complex and the “IT-based” approach was able to produce better results. The application of the software in this outbreak investigation confirmed the following advancements compared to traditional methods:

- supply chains could be fully reconstructed in terms of individual lots
- visualization and analysis could be done automatically
- analysis could be restricted to lots that have been processed in specific time frames
- amounts of incoming commodities could be verified against outgoing amounts at each station to identify gaps in data
- local investigations could be minimized; e.g. software-based analysis showed that there was no matching lot-related linkage for a seed distributor who had supplied all outbreak clusters
- the common source linking German and French cases was first identified by the software (Fig 6)

In 2013/2014 FoodChain-Lab has been used by an EFSA working group investigating a hepatitis A outbreak in Europe [10]. It was clear from initial outbreak investigations that the food commodities contaminated with hepatitis A virus were berries. It was crucial for risk management purposes to identify the berry type, the crop season, and the country or region of origin. The working group therefore collected additional lot-based data from affected EU member states to perform a more detailed tracing investigation.

The application of FoodChain-Lab in this investigation demonstrated that:

- outbreaks with large volumes of data could be handled
- investigators were more motivated to collect local information
- software simulation and visualization functionalities facilitated generation of new hypotheses and data exploration
- a ranking of most likely scenarios and stations/deliveries of interest could be generated
- berry type and crop season could be analyzed separately
- traces could be checked for having reached the primary producer
- regional analysis enabled identification of a specific geographical area (not described by administrative boundaries or otherwise defined) as the potential common origin of contaminated foods, which would have been impossible to identify using traditional methods

As in both real world applications dirty or incomplete data was the rule rather than the exception, the most valuable software features were data integration with plausibility checks, data cleaning and detection of missing data, allowing quicker, less error-prone, semi-automated and self-explanatory visual data analysis to support risk and outbreak managers.

Applications in real world outbreak investigations allowed iterative technical improvements to the software using expertise from software engineers, epidemiologists, risk assessors, food microbiologists and veterinarians.

## Discussion and Forward Look

FoodChain-Lab is a software tool designed to support the investigation of food-related outbreaks. Traceback analyses can be useful to generate hypotheses of the source of the outbreak. Stations or lots on the trace of the outbreak clusters with higher tracing scores require further investigation, for example via on-site microbiological examinations. Traceforward analysis is also supported by FoodChain-Lab and may identify unknown outbreak clusters or cases or stations where contaminated food commodities might still be available.

FoodChain-Lab was found to be of value during past foodborne outbreaks. However, we identified additional requirements to facilitate and enhance analysis of outbreak investigations. For example, a standardized information exchange format for tracing data would speed up investigations significantly. As long as there is no such standard FoodChain-Lab will be successively developed further to support existing formats on demand. Another improvement for data collection would be a centralized and web-based data management infrastructure giving direct automated feedback on data quality to the data provider. The next developmental step for FoodChain-Lab will be a semi-automatic hypothesis generation feature permitting the automatic exploration of contamination sources with high scores by simulating cross-contamination, stop-contamination and regional aggregation of stations and lots.

Other features potentially enhancing outbreak investigations are:

- Integration of information collected from mobile devices, as has been explored in the field of epidemiological investigation. There, exposed persons have been interviewed using smart phones which proved to increase response rates and speed up data collection considerably [26].
- Integration with alternative outbreak investigation approaches such as the likelihood-based approach reported to be applicable for the identification of contaminated food products under certain conditions. This approach compares the spatial distribution pattern of case reports with the sales data-based distribution pattern of food products under suspicion. This approach could be applicable if the outbreak has been caused by a single food product sold and consumed at multiple locations [27].
- Integration of other software tools that are already designed to interact with FoodChain-Lab such as PMM-Lab [28] and FoodProcess-Lab [29]. These tools provide modelling features in the field of predictive microbiology and share the same database as FoodChain-Lab. If more details on the processing of products can be gathered by local authorities, e.g. environmental conditions regarding storage or production, it will be possible to import any such data into the database. The modelling tools can perform the calculation of the survival probabilities of agents within food processing stations. This will finally influence the calculation of the Tracing Score.
- Integration of epidemiological data and models (e.g. a SIR model) enhancing exposure and risk assessments for forward tracing scenarios. This could be done by integrating the Spatiotemporal Epidemiological Modeler Project [30].

The software described in this article illustrates which benefits can be achieved if knowledge from different disciplines is combined freely and information is exchanged to the highest extent possible in multidisciplinary teams. Strengthening the collaboration between competent authorities and food business operators, e.g. by agreeing on and using common data formats for traceability purposes would contribute toward food safety and limiting foodborne outbreaks.

## References

[1] Hoffmann S, Batz MB, Morris JG. Annual cost of illness and quality-adjusted life year losses in the United States due to 14 foodborne pathogens. J Food Prot [Internet]. 2012 7 [cited 2014 Sep 26];75(7):1292–302. Available from: http://www.ncbi.nlm.nih.gov/pubmed/22980013 10.4315/0362-028X.JFP-11-417 22980013

[2] Gadiel D. The economic cost of foodborne disease in New Zealand. New Zeal Food Saf Auth 2010;

[3] Abe K, Yamamoto S, Shinagawa K. Economic impact of an Escherichia coli O157:H7 outbreak in Japan. J Food Prot [Internet]. 2002 1;65(1):66–72. Available from: http://www.ncbi.nlm.nih.gov/pubmed/11808808 10.4315/0362-028x-65.1.66 11808808

[4] Dwyer DM, Strickler H, Goodman R a., Armenian HK. Use of case-control studies in outbreak investigations. Epidemiol Rev. 1994;16:109–23. 7925720 10.1093/oxfordjournals.epirev.a036137

[5] Regattieri A, Gamberi M, Manzini R. Traceability of food products: General framework and experimental evidence. J Food Eng. 2007 7;81(2):347–56.

[6] Regulation No 178/2002 of the European Parliament and of the Council of 28 January 2002 [Internet]. Available from: http://eur-lex.europa.eu/LexUriServ/LexUriServ.do?uri=OJ:L:2002:031:0001:0024:EN:PDF

[7] Weiser AA, Gross S, Schielke A, Wigger J-F, Ernert A, Adolphs J, et al Trace-back and trace-forward tools developed ad hoc and used during the STEC O104:H4 outbreak 2011 in Germany and generic concepts for future outbreak situations. Foodborne Pathog Dis [Internet]. 2013 3 [cited 2014 Jan 5];10(3):263–9. Available from: http://www.pubmedcentral.nih.gov/articlerender.fcgi?artid=3698685&tool=pmcentrez&rendertype=abstract 10.1089/fpd.2012.1296 PMC3698685 23268760

[8] Anonymous. Großer Gastroenteritis-Ausbruch durch eine Charge mit Noroviren kontaminierter Tiefkühlerdbeeren in Kinderbetreuungseinrichtungen und Schulen in Ostdeutschland. Epidemiol Bull. 2012;41:414–7.

[9] EFSA. Request to EFSA on scientific assistance in a multinational outbreak of Hepatitis A [Internet]. 2013. Available from: http://registerofquestions.efsa.europa.eu/roqFrontend/questionLoader?question=EFSA-Q-2013-00878

[10] European Food Safety Authority. Tracing of food items in connection to the multinational hepatitis A virus outbreak in Europe. 2014;12(9):1–186.

[11] Levenshtein V. Binary codes capable of correcting deletions, insertions, and reversals. Sov Phys Dokl. 1966;10(8):707–10.

[12] O’Madadhain J, Fisher D, Smyth P. Analysis and visualization of network data using JUNG. J Stat Softw. 2005;VV(Ii):1–35.

[13] Fruchterman TMJ, Reingold EM. Graph drawing by force-directed placement. Softw Pract Exp [Internet]. 1991 11 [cited 2014 Oct 13];21(11):1129–64. Available from: http://doi.wiley.com/10.1002/spe.4380211102

[14] ESRI. ESRI Shapefile Technical Description. Esri White Pap [Internet]. 1998 [cited 2014 May 19]; Available from: http://scholar.google.com/scholar?hl=en&btnG=Search&q=intitle:ESRI+Shapefile+Technical+Description#1

[15] OpenStreetMap [Internet]. Available from: http://openstreetmap.org

[16] MapQuest. MapQuest Open Geocoding API Web Service [Internet]. Available from: http://developer.mapquest.com/web/products/open/geocoding-service

[17] Masclet D. Gisgraphy [Internet]. 2014. Available from: http://www.gisgraphy.com

[18] MacQueen J. Some Methods for classification and analysis of multivariate observations. Proc Fifth Berkeley Symp Math Stat Probab. 1967;1:281–97.

[19] Ester M, Kriegel H-P, Sander J, Xu X. A Density-Based Algorithm for Discovering Clusters in Large Spatial Databases with Noise. Proceedings of the Second International Conference on Knowledge Discovery and Data Mining. 1996. p. 226–31.

[20] Berthold MR, Cebron N, Dill F, Gabriel TR, Kötter T, Meinl T, et al KNIME—the Konstanz information miner. ACM SIGKDD Explor Newsl. 2009 11 16;11(1):26.

[21] Riden CP, Bollen AF. Agricultural supply system traceability, Part II: Implications of packhouse processing transformations. Biosyst Eng [Internet]. 2007 12 [cited 2015 Dec 9];98(4):401–10. Available from: http://www.sciencedirect.com/science/article/pii/S1537511007001821

[22] Dupuy C, Botta-genoulaz V, Guinet A. Traceability analysis and optimization method in food industry. IEEE International Conference on Systems, Man and Cybernetics. IEEE; 2002. p. 494–9.

[23] Donnelly KAM, Karlsen KM, Olsen P, Van Der Roest J. Creating standardised data lists for traceability: a study of honey processing. Int J Metadata, Semant Ontol. 2008 1 1;3(4):283.

[24] LeBlanc DI, Villeneuve S, Hashemi Beni L, Otten A, Fazil A, McKellar R, et al A national produce supply chain database for food safety risk analysis. J Food Eng [Internet]. 2015 2 [cited 2015 Dec 9];147:24–38. Available from: http://www.sciencedirect.com/science/article/pii/S026087741400394X

[25] Berichte zur Lebensmittelsicherheit 2012: Bundesweiter Überwachungsplan 2012. Springer-Verlag; 2013 57 p.

[26] Simone B, Atchison C, Ruiz B, Greenop P, Dave J, Ready D, et al Investigating an outbreak of Clostridium perfringens gastroenteritis in a school using smartphone technology, London, March 2013. Eurosurveillance [Internet]. European Centre for Disease Prevention and Control (ECDC)—Health Comunication Unit; 2014 5 15 [cited 2014 Oct 13];19(19):16–22. Available from: http://www.eurosurveillance.org/ViewArticle.aspx?ArticleId=20799 10.2807/1560-7917.es2014.19.19.20799 24852955

[27] Kaufman J, Lessler J, Harry A, Edlund S, Hu K, Douglas J, et al A Likelihood-Based Approach to Identifying Contaminated Food Products Using Sales Data: Performance and Challenges. PLoS Comput Biol. Public Library of Science; 2014;10(7):e1003692. 10.1371/journal.pcbi.1003692 PMC4080998 24992565

[28] Filter M, Thöns C, Brandt J, Weiser AA, Falenski A, Appel B, et al. A community resource for integrated predictive microbial modelling (PMM-Lab). 5th International Workshop Cold Chain Management. 2013.

[29] Weiser AA, Falenski A, Thöns C, Filter M. FoodProcess-Lab [Internet]. 2014. Available from: https://sourceforge.net/projects/foodprocesslab/

[30] Kaufman JH, Davis M, Douglas J, Edlund S. The SpatioTemporal Epidemiological Modeler [Internet]. 2014. Available from: http://www.eclipse.org/stem/

